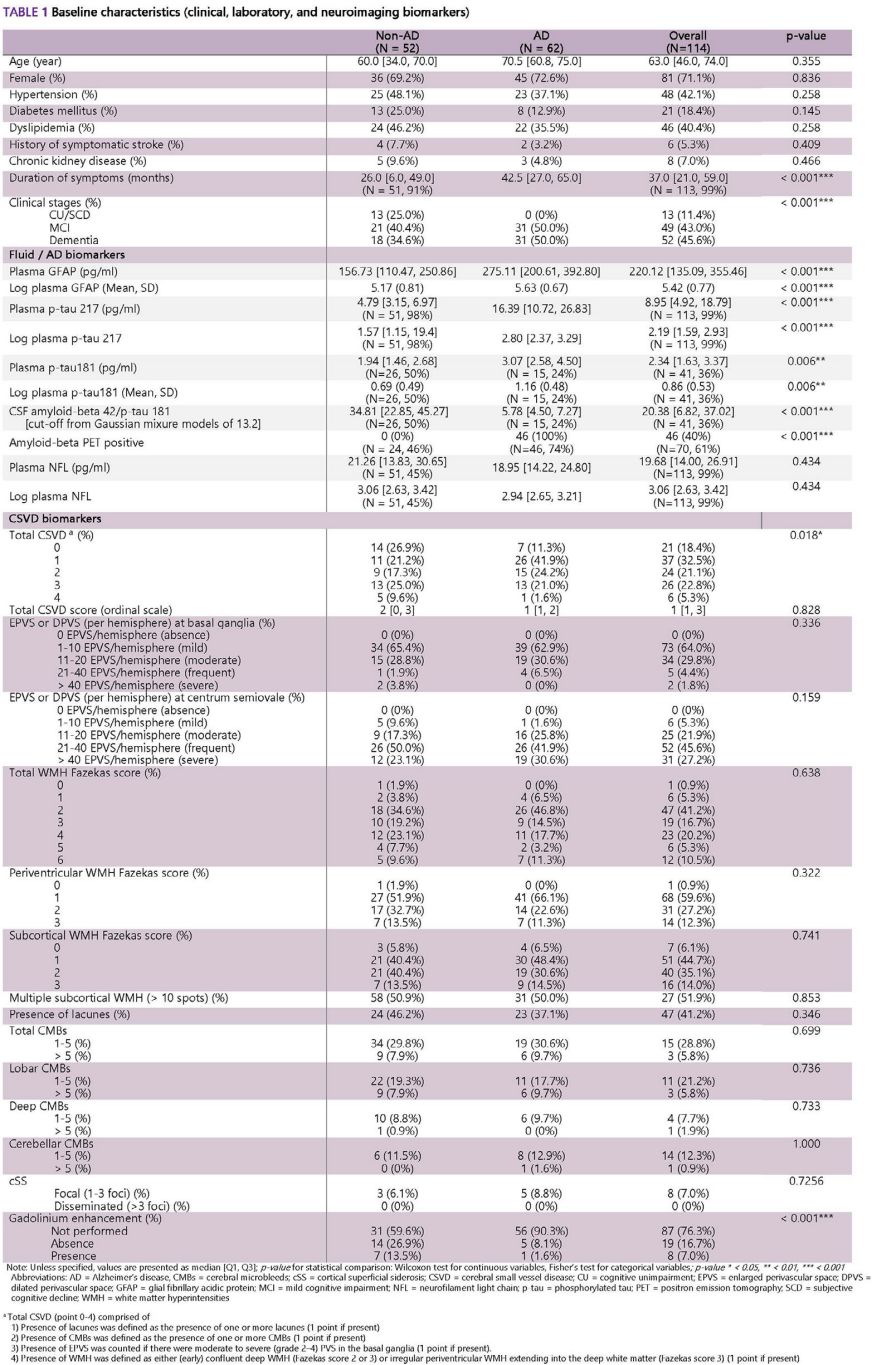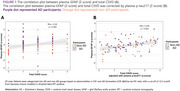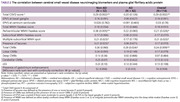# Correlation between cerebral small vessel disease biomarkers and plasma GFAP in participants of the encephalopathy and memory clinic cohort, in Thailand: A Story from a Middle‐Income Country

**DOI:** 10.1002/alz70856_098874

**Published:** 2025-12-24

**Authors:** Thanakit Pongpitakmetha, Thanapoom Taweephol, Nattanich Pornteparak, Juthamas Rianaree, Tatchaporn Ongphichetmetha, Thachamai Smitasiri, Sekh Thanprasertsuk, Adipa Chongsuksantikul, Prawit Oangkhana, Watayuth Luechaipanit, Thanaporn Haethaisong, Jedsada Khieukhajee, Kittithatch Booncharoen, Yuttachai Likitjaroen, Poosanu Thanapornsangsuth

**Affiliations:** ^1^ Department of Pharmacology, Faculty of Medicine, Chulalongkorn University, Bangkok, Thailand; ^2^ Division of Neurology, Department of Medicine, Faculty of Medicine, Chulalongkorn University, Bangkok, Thailand; ^3^ Chula Neuroscience Center, King Chulalongkorn Memorial Hospital, The Thai Red Cross Society, Bangkok, Thailand; ^4^ Memory Clinic, King Chulalongkorn Memorial Hospital, The Thai Red Cross Society, Bangkok, Thailand; ^5^ Department of Microbiology, Faculty of Medicine, Chulalongkorn University, Bangkok, Thailand; ^6^ Department of Medicine, Banphaeo General Hospital, Samutsakhon, Thailand; ^7^ Siriraj Neuroimmunology Center, Faculty of Medicine Siriraj Hospital, Mahidol University, Bangkok, Thailand; ^8^ Department of Physiology, Faculty of Medicine, Chulalongkorn University, Bangkok, Thailand; ^9^ Chula Neuroscience Center, King Chulalongkorn Memorial Hospital, Bangkok, Thailand; ^10^ Cognitive, Clinical and Computational Neuroscience (CCCN) Center of Excellence, Chulalongkorn University, Bangkok, Thailand; ^11^ Thai Red Cross Emerging Infectious Diseases Health Science Centre, King Chulalongkorn Memorial Hospital, Bangkok, Thailand; ^12^ Thai Red Cross Emerging Infectious Diseases Health Science Centre, King Chulalongkorn Memorial Hospital, The Thai Red Cross Society, Bangkok, Thailand; ^13^ Neurological Institute of Thailand, Ratchathewi, Bangkok, Thailand; ^14^ Neurology Center, Phyathai 1 Hospital, Bangkok, Rachathewi, Thailand; ^15^ Neurocognitive Unit, Division of Neurology, Department of Medicine, Faculty of Medicine, Chulalongkorn University, Bangkok, Thailand

## Abstract

**Background:**

Cerebral small vessel disease (CSVD) is one of the leading causes of vascular cognitive impairment and dementia (VCID) in the aging population. It may also contribute to a faster rate of clinical deterioration in Alzheimer's disease (AD) as a co‐pathology. Plasma glial fibrillary acidic protein (GFAP) may be elevated due to astrocytosis in either CSVD or AD pathogenesis. This study evaluates the correlation between CSVD neuroimaging biomarkers and plasma GFAP in a memory clinic cohort.

**Methods:**

The clinical information and plasma biomarkers were collected from memory clinic cohorts. Patients were categorized into AD and non‐AD groups based on either an abnormal cerebrospinal fluid Amyloid beta (Aβ) 42/p‐tau181 ratio, or a positive Aβ Positron Emission Topography. Magnetic resonance imaging (MRI) was independently rated by the two trained investigators using STRIVE‐2 criteria *(Duering M, 2023)*. The validated composite total CSVD score *(Staals J, 2014)* ranging from 0‐4 was calculated. The non‐parametric statistical analysis was applied to analyze.

**Results:**

A total of 114 participants were enrolled. Clinical information, laboratory test results, and neuroimaging biomarkers were compared between AD and non‐AD groups, as shown in Table 1. A positive correlation exists between the total CSVD score and plasma GFAP in the whole and non‐AD cohort (Spearman rho = 0.29, *p*‐value 0.002). After the adjustment for plasma *p*‐tau 217, the notable positive correlation between total CSVD score and plasma GFAP in the whole cohort (Spearman rho = 0.56, *p*‐value < 0.001) is observed as demonstrated in visualized correlation plots in Figure 1. The results of Spearman's correlation and the Wilcoxon rank sum test between plasma GFAP and each neuroimaging biomarker are summarized in Table 2, where only certain neuroimaging biomarkers demonstrated a positive correlation.

**Conclusion:**

The total CSVD score and plasma GFAP showed a stronger positive correlation in the non‐AD group than in the AD group. After adjusting for *p*‐tau 217, an AD biomarker, the correlation between total CSVD score and plasma GFAP became stronger. These findings suggest that plasma GFAP alone may have limitations in assessing CSVD burden in AD due to shared mechanisms and biomarkers. Further validation of other potential VCID fluid biomarkers is warranted.